# Supplementary material for: Considerable Variation in Intake of Live Food Microbes in Dutch Adults
Source: Nutrients. 2025 Apr 3;17(7):1248. doi: 10.3390/nu17071248 (PMC11990227; doi:10.3390/nu17071248)
Supplement: Supplementary file 1 [file nutrients-17-01248-s001.zip › nutrients-3537415-supplementary.pdf]

Section S1

Table S1. Food Microbial Load Table.

	Total Contaminating Bacteria TCB) (CFU/g)			*Lactobacillus* (LAB) (CFU/g)			Yeasts/Moulds (YM) (CFU/g)		
	Min	Best	Max	Min	Best	Max	Min	Best	Max
**Potatoes**									
Potatoes	0	10^1	10^3						
**Vegetables**									
Vegetables raw	10^2	10^6	10^9						
Vegetables cut (raw)	10^2	10^6	10^9						
Vegetables cooked	0	10^1	10^3						
Vegetables stir fried	10^2	10^4	10^6						
Vegetables (glass/canned)	0	10^1	10^2						
Sauerkraut (raw) *				10^6	10^7	10^9			
Pickles *							0	10^2	10^5
Pickled onion *							0	10^2	10^5
Mushrooms *	10^5	10^7	10^8				0	10^4	10^8
Sprouting (raw)	10^5	10^7	10^9						
Legumes	0	10^1	10^3						
**Fruit and nuts**									
Fruit (with peel)	10^1	10^2	10^7				10^1	10^3	10^7
Fruit (without peel)	10^2	10^3	10^7				10^2	10^4	10^7
Fruit salad (excl. canned)	10^2	10^3	10^7				10^2	10^4	10^7
Fruit (canned)							0	10^1	10^2
Fruit dried							10^1	10^3	10^4
Nuts, Seeds, Nut spread							10^1	10^3	10^4
Mixed fruit/nuts							10^1	10^3	10^4
Olives				10^5	10^6	10^8			
**Dairy products**									
Milk	0	10^4	10^7	0	10^4	10^7			
Milk raw	10^4	10^6	10^8						
Milk Drink				0	10^4	10^7			
Buttermilk				10^6	10^7	10^9			
Yoghurt *				10^6	10^8	10^9			
Yoghurt Drink				10^6	10^7	10^8			
Quark, fresh cheese				10^6	10^8	10^9			
Cheese (excl. cheese with moulds)				10^6	10^8	10^9			
Cheese with moulds				10^5	10^7	10^9	10^5	10^7	10^8
Pudding, Custard	0	10^4	10^6						
Cream and cream replacers	0	10^4	10^6						
Coffee milk and –creamers	0	10^3	10^6	0	10^3	10^6			
**Grain and grain products**									
Flour, wheat, etc							10^2	10^3	10^4
Pasta, rice							0	10^1	10^3
Bread							10^1	10^3	10^4
Crisp bread, Rusk							0	10^1	10^2

	Total Contaminating Bacteria TCB) (CFU/g)			*Lactobacillus* (LAB) (CFU/g)			Yeasts/Moulds (YM) (CFU/g)		
	Min	Best	Max	Min	Best	Max	Min	Best	Max
Rye bread *				0	0	10^{6}	0	10^{3}	10^{4}
Muesli	10^{2}	10^{4}	10^{6}				10^{2}	10^{3}	10^{4}
Cornflakes	10^{2}	10^{3}	10^{4}				10^{2}	10^{3}	10^{4}
"Brinta" (instant wheat flakes)	10^{1}	10^{2}	10^{3}				10^{1}	10^{2}	10^{3}
Other breakfast cereals							10^{2}	10^{3}	10^{4}
Chips							0	10^{1}	10^{2}
**Meat**									
Meat (baked/cooked)	0	10^{2}	10^{4}						
Meat (canned)	0	10^{1}	10^{2}						
Cold cut fermented *				10^{1}	10^{7}	10^{9}			
"Zure Zult" (Vinegared brawn)							10^{1}	10^{4}	10^{6}
Cold cut (remaining)	10^{1}	10^{5}	10^{8}	10^{1}	10^{5}	10^{9}			
**Fish**									
Fish (canned)	0	10^{1}	10^{2}						
Fish raw	10^{4}	10^{7}	10^{8}						
Fish baked	0	10^{1}	10^{2}						
Fish smoked/steamed	10^{1}	10^{3}	10^{4}						
Shellfish, shrimps	10^{2}	10^{4}	10^{7}						
Fish products	0	10^{1}	10^{2}						
**Eggs**									
Eggs	0	10^{1}	10^{2}						
**Fats and oils**									
Fats, non-specific							0	10^{2}	10^{3}
Oil vegetable							0	10^{2}	10^{3}
Butter							0	10^{1}	10^{5}
Margarine							0	10^{1}	10^{5}
**Sugar, candy and sweet bread filling**									
Sugar, jam, honey	10^{1}	10^{2}	10^{3}				0	10^{1}	10^{2}
Chocolate, chocolate bars							0	10^{2}	10^{3}
Confectionery							0	10^{2}	10^{3}
Ice cream, popsicle	10^{1}	10^{4}	10^{6}						
Dessert sauce							0	10^{1}	10^{2}
**Cookies, pastries and cakes**									
Gingerbread	10^{1}	10^{3}	10^{4}				0	10^{2}	10^{3}
Pastries	10^{2}	10^{5}	10^{6}				10^{1}	10^{2}	10^{3}
Biscuits, cookies, cake							0	10^{2}	10^{3}
**Non-alcoholic beverages**									
Fruit juice	10^{2}	10^{4}	10^{5}				10^{1}	10^{4}	10^{5}
Vegetable juice	10^{2}	10^{4}	10^{5}				10^{1}	10^{4}	10^{5}
Soft drinks, sport drinks							10^{1}	10^{2}	10^{3}
Coffee, tea							0	10^{1}	10^{2}
Water	0	10^{1}	10^{2}						
**Alcoholic beverages**									
Beer *				0	0	10^{6}	0	0	10^{6}
**Sauces**									
Sauce warm (excl. gravy)	10^{1}	10^{3}	10^{5}				0	10^{2}	10^{3}

	Total Contaminating Bacteria TCB) (CFU/g)			*Lactobacillus* (LAB) (CFU/g)			Yeasts/Moulds (YM) (CFU/g)		
	Min	Best	Max	Min	Best	Max	Min	Best	Max
Gravy		10^2	10^3				0	10^2	10^3
Mayonnaise, Ketchup	0	10^2	10^3				0	10^2	10^3
Soy sauce *	0	10^2	10^3				0	10^2	10^3
Herbs and spices	10^4	10^6	10^7						
"Sandwich spread"				10^1	10^3	10^7	10^1	10^3	10^5
Savory salad *				10^2	10^4	10^5	10^2	10^4	10^5
**Soups**									
Soup	10^2	10^4	10^5						
Bouillon	10^2	10^4	10^5						
**Other**									
Soya yoghurt *				0	10^6	10^8			
Vegetarian meat replacer (bread) *	0	10^5	10^7	0	10^5	10^7			
Vegetarian meat replacer *	0	10^2	10^3						
Yeast							10^1	10^4	10^6
Marmite							10^1	10^2	10^3
Snacks (savoury)	10^1	10^3	10^4						
Warm meals average	10^2	10^4	10^6						
Cold meals (uncooked)	10^2	10^6	10^8	10^2	10^4	10^6			

* = Products that were tested in the laboratory by us.

Section S2. Determination of Microbial Content of Duplicate Foods

All steps of sample analyses were performed in an aseptic way. Frozen and homogenized samples were defrosted within 3 hours at room temperature. From every sample ten gram was weighted out into a stomacher bag. The sample was diluted 1:10 with Peptone Physiological Salt Solution (PPS), using a Delta Diluter (IUL Instruments) and blended using a Stomacher® 400 circulator. From this primary dilution all necessary dilutions were made. For the *Lactobacillus* count and the aerobic mesophilic plate count the pour plate method was used. Pour plates were prepared by adding 1 ml of the required dilution to a sterile Petri dish after which ±15 ml of the appropriate growth medium (tempered to 46 ± 1°C) was added.

Lactobacillus count was determined using de Man Rogosa Sharpe Agar (MRSA, Merck/Oxoid) as medium. Plates were incubated for 3 days at 30°C. Total aerobic bacteria count (TAB) was determined with Plate Count Agar (PCA, Oxoid), a non-selective growth medium. Plates were incubated for 3 days at 30°C. It should be noted that *Lactobacillus* is also often able to grow on PCA. The spiral plate count method was used to determine fungi count. Oxytetracycline Glucose Yeast Extract Agar (OGYE, Oxoid) was used as medium. The spiral plater used was an Eddy Jet Spiral Plater (IUL Instruments). Plates were incubated for 5 days at 25°C.

For the *Lactobacillus* (LAB) a confirmation test was done using a catalase test with hydrogen peroxide (3%). First a minimum of three colonies were streaked onto a MRSA plate, which was then incubated at 30°C for 24 hours under micro-aerobic conditions. After incubation a drop of hydrogen peroxide solution was placed onto an object glass. Using a sterile loop, material from the edge of a colony was taken and put inside the hydrogen peroxide solution. If gas was formed, it meant that the test was positive. If the test was positive, it meant that LAB was not confirmed.

To calculate the daily microbial exposure, the CFU's g^{-1} was multiplied by the total weight of the subject's collected food.

Section S3. Calculation of Contribution of Foods to the Variance in Microbial Exposure

Variance, measured as the R^2_w value, resulted from the variance of one particular food relative to the variance that was explained by consuming all foods in the list [32]. In this study, the variance of microbial exposure was calculated for both the best and the maximum estimations.

$$\text{Contribution to microbial exposure} = \left(\bar{\hat{Z}}/\bar{Z}\right) \times 100\ \%$$

$$\text{Contribution to variance} = R^2_w = \sum_{i=1}^{n}\left(\hat{Z}_i - \bar{\hat{Z}}\right)^2 \Bigg/ \left(\sum_{i=1}^{n}\left(Z_i - \bar{Z}\right)^2\right) \times 100\ \%$$

R^2_w: variance resulted from consumption of selected food

Z_i: total dietary microbial exposure of one person as a result of consuming all food groups in the list (CFU/day)

$\bar{Z}$: mean of total dietary microbial exposure in the study population as a result of consuming all food groups in the list

$\hat{Z}$: total dietary microbial exposure of an individual in the study population as a result of consuming one particular food group (CFU/day)

$\bar{\hat{Z}}$: Mean of individual total dietary microbial exposure in the study population as a result of consuming one particular food group (CFU/day)

Contribution to the level of exposure was defined as microbial exposure gained from consuming one food relative to the total exposure from consuming all foods in the list.
